# Qualitative inquiry into adolescents’ experience of ethical challenges during enrollment and adherence to antiretroviral therapy (ART) in Temeke Regional Referral Hospital, Tanzania

**DOI:** 10.1186/s12910-022-00762-3

**Published:** 2022-03-09

**Authors:** Renatha Sillo Joseph, Gladys Reuben Mahiti, Gasto Frumence, Connie M. Ulrich

**Affiliations:** 1 Department of Bioethics and Health Professionalism, School of Public Health and Social Sciences, Muhimbili University of Health and Allied Sciences, Dar es Salaam, Tanzania; 2 Department of Developmental Studies, School of Public Health and Social Sciences, Muhimbili University of Health and Allied Sciences, Dar es Salaam, Tanzania; 3 Biobehavioral Department, School of Nursing, Department of Medical Ethics and Health Policy, and New Courtland Center for Transitions and Health, University of Pennsylvania, Philadelphia, PA USA

**Keywords:** Adolescents, Ethical issues, HIV care and treatment, Adherence to antiretroviral therapy

## Abstract

**Background:**

Adolescents living with human immunodeficiency virus (HIV) experience challenges, including lack of involvement in their care as well nondisclosure of HIV status, which leads to poor adherence to antiretroviral therapy (ART). Parents have authority over their children, but during adolescence there is an increasing desire for independence. The aim of the study was to explore adolescents’ experience of challenges identified by adolescents ages 10–19 years attending HIV care and treatment at Temeke Regional Referral Hospital in Tanzania.

**Methods:**

An exploratory descriptive qualitative design was employed in the HIV Care and Treatment Centre (CTC) in the Out-Patient Department at the Temeke Regional Referral Hospital in Tanzania with adolescents living with HIV who were 10–19 years of age. A total of 22 adolescents participated in semi-structured face-to-face interviews after parental consent and adolescent assent were obtained. Participants were interviewed about their participation in decisions to be tested for HIV and enrolled in the CTC, concerns surrounding disclosure of their HIV status to the adolescent or to others, stigma and discrimination, and the effect of these challenges on their adherence to medication. All interviews were audio-taped, transcribed verbatim in Swahili, and back-translated to English. Data analysis included both inductive and deductive thematic analysis.

**Results:**

Qualitative themes identified included lack of participation in decisions about HIV testing, challenges to enrollment in care and treatment; issues around disclosure of HIV status, such as delays in disclosure to the adolescent and disclosure to other persons and benefits and harms of such disclosures; and factors supporting and interfering with adherence to ART, such as parental support, organizational (clinic) support and problems, and self-stigmatization and shame.

**Conclusion:**

Lack of adolescents’ involvement in their care decision making and delayed disclosure of HIV status to the adolescent were identified concerns, leading to poor adherence to ART among adolescents. Disclosure to others, especially teachers, helped adolescents at school to take their medication properly. Disclosure to others led to stigma and discrimination for some adolescents. More research is needed to better understand the role of disclosure and its benefits and challenges for HIV-positive adolescents in Tanzania.

## Background

Adolescence is defined by the World Health Organization as a time of transition from childhood to adulthood for those persons 10–19 years of age [1]; it is also a unique developmental period when capacity for decision making evolves [2]. Indeed, adolescents have an increasing need to be involved in making decisions related to their care and well-being [3, 4]. Parents should foster adolescents’ participation in decision making that supports their ability to make informed judgments [5]. Adolescent involvement in their own health care decision making can create a positive relationship between the doctor, the patient, and the parent, enhancing adolescents’ satisfaction with their care and trust between all parties [6]. Even though adolescents should participate in decisions that affect their health and well-being, parents have the authority to make decisions for their sons and daughters [7].

When an adolescent is ill, parents and the adolescent may disagree about what should be done or whose decision should be followed [8]. Adolescents’ growing desire for independence may lead to conflict between them and their parents or designated care givers [9]. This is the case, for example, when an adolescent has a diagnosis of HIV and/or acquired immunodeficiency syndrome (AIDS) and ethical issues often arise in the African setting [10].

Adolescence is a time when decisions may need to be made about HIV testing and/or disclosure of previously known HIV status to the adolescent. Indeed, nearly 24% of the Tanzanian population in 2020 were adolescents aged 10–19 years [11]. In 2019, 11% of Tanzanian children were born with HIV infection via perinatal transmission [12]. Many Tanzanian parents who are aware of their infant’s HIV infection choose not to tell the young child, and instead administer daily medications throughout childhood. There are many challenges regarding continued nondisclosure of HIV status to adolescents living with HIV. HIV disclosure to adolescents is often surrounded by parents’ fear of emotionally distressing a child [13]. Some adolescents infected perinatally may not know their HIV status; when there is no proper disclosure and sex education, these adolescents may infect others [14]. In fact, a qualitative study at Mbagala tertiary health facility in Tanzania found low rates of HIV status disclosure to children and adolescents under age 15 [15]. Low rates of HIV disclosure may lead to poor antiretroviral therapy (ART) adherence and other ethical concerns.

Due to the adolescent’s developing capacity to understand, adolescence is also a time when individuals enroll in HIV care and treatment programs in Tanzania learn about the importance of adherence to care. The HIV Care and Treatment Centre (CTC) in the Out-Patient Department at the Temeke Regional Referral Hospital in Tanzania is a health care facility that provides care and treatment for individuals living with HIV and AIDs; it was implemented in 2004 [16]. Ensuring adherence to ART among adolescents can present unique challenges. A qualitative study conducted in Canada with 34 HIV-positive young people documented their confusion and misunderstanding of HIV treatment; some felt they had no choice about treatment, some cited disruptions to their lives, and some reported difficulties in adhering to ART [17]. Many factors may influence adolescents’ adherence to ART, including their motivations to receive care, the serious nature of their illness, communication styles in the clinical interaction, peer support, and structural issues [18] and whether they know their HIV status [19], among other issues.

Risky behavior and curiosity expose adolescents to the potential for unprotected sex and increased risk of HIV infection [20]. In Tanzania, adolescents’ enrollment in care and treatment for HIV and AIDS is low [21]. On top of the low enrollment, there is also a high rate of loss to follow-up and poor adherence to medication, which further increase HIV infection incidence and mortality. Thus, there is a great need to understand the challenges, including the ethical concerns that adolescents face because of their HIV status. The purpose of this study was to understand adolescents' experience of their HIV-positive challenges that influenced their adherence to ART among adolescents attending an HIV care and treatment facility in Temeke Regional Referral Hospital, Tanzania.

## Methods

An exploratory, qualitative, descriptive design was used to examine adolescents’ experience of HIV-positive challenges that influenced their enrollment in CTC and adherence to ART at Temeke Regional Referral Hospital, in Dar es Salaam, Tanzania**.** This design was appropriate, first to gain knowledge concerning the challenges and ethical concerns that influence adolescents to enroll in and adhere to treatment within the African context, and second to describe the events and experiences of adolescents at Temeke CTC [22]. Adolescents participated in semi-structured interviews, as described below.

### Ethical considerations

Ethical clearance was obtained from Muhimbili University of Health and Allied Sciences (MUHAS) Institutional Review Board and the National Research Ethics Review Committee. Written informed consent and pediatric assent were obtained from parents with adolescents ages 10–17 years [1]. Older adolescents (18- and 19-year-olds) consented on their own.

### Data collection

This qualitative study was part of a larger quantitative study to assess knowledge of ethical issues among adolescents living with HIV and their parents and health care providers. Data were collected through in-depth semi-structured interviews conducted in a private location in the clinic. The interview guide was developed from a literature review of ethical issues that influence adherence to ART among adolescents and in consultation with qualitative experts on the study team [23–26]. Prior to full data collection, a small pilot study involving four adolescents was conducted at Amana Regional Referral Hospital in Tanzania. This site was selected because it is similar to the CTC at Temeke Regional Referral Hospital. Minor adjustments were made to several interview questions for clarity. Research assistants were trained on interviews, data collection tools, and transcription before going to Temeke CTC. Research assistants were two post graduate students at MUHAS, and they assisted the principal investigator (PI) in conducting interviews and transcribing audio-taped interviews.

Parents of adolescents with an appointment at the clinic were phoned by a “leader” of the adolescents to confirm their appointments. These leaders are adolescents selected to lead others in clinic activities, and their role is to assist in setting appointments for their peer group and to schedule follow-up appointments for those who did not show up for their initial appointment. The PI worked with these leaders to phone adolescents who were on the list for the day to come with their parents to ask for their permission to participate in the study. On arrival, the PI introduced parents to the study and asked them if they would be interested in participating. A total of 28 were approached, and six declined; five parents declined on behalf of their adolescent, and one adolescent personally declined. Purposeful sampling was used to select participants based on their age [10–19], educational level (primary school, secondary school, vocational education and training authority students, and university students), ability to express themselves, knowledge of their positive HIV status, and enrollment in ART. During the interviews, which were conducted by research assistants and the PI, parents or those designated as primary care givers who were not biological parents waited outside the interview room. Saturation was achieved with 22 adolescents, when data analysis and review indicated that no new themes or ideas were expressed by participants.

The interview guide and interviews were in Swahili—the national language comfortable to all participants. The average duration of each interview was 25 min. The interview guide provided open-ended questions to solicit participants’ descriptions of their experiences of challenges and ethical concerns during CTC attendance. Participants described how such challenges influenced their enrollment in CTC and adherence to ART. Probing questions were asked in areas that needed more clarification or elaboration. Interviews were audiotaped with participants’ consent and transcribed verbatim; transcripts were translated to English. The study was conducted from January to August 2020. Although data collection was expected to be completed at the end of March 2020, there was a slight delay due to the COVID-19 pandemic, but the study was able to resume in July and completed as indicated.

### Data analysis and qualitative rigor

Descriptive statistics were used to analyze participants’ demographic characteristics, including age, gender, duration of ART, age at enrollment in the CTC, and age when the individual learned of his/her HIV status. After transcription, each transcript was uploaded to NVivo 12 software, which assisted in data coding. Immediately following the interview, open coding was conducted on printed hard copies of the transcript, before the transcript was uploaded to the software. We used the principles of thematic analysis to explore adolescents’ experience of their challenges during HIV testing, attending the CTC, and using ART [27]. Thematic analysis helps researchers identify and examine themes or patterns within a particular context.

Six steps for thematic analysis were followed [22]. First, all transcripts were reviewed by the PI and co-investigator to assess validity of the data and become familiar with the data. Second, phrases, sentences, and paragraphs were organized in a meaningful way to form codes. We used open coding; codes were developed and modified as the coding process progressed. Third, we examined related codes, and codes were then refined into categories. Some of them were collated into initial themes. At the end of this step, the codes were organized into broader themes that seemed to specifically describe issues in adolescents’ HIV care and treatment. Fourth, themes were reviewed to identify whether they were supported by the data. Themes that were related or seemed to address the same issue or concern were combined to form a single theme, and those that were not supported by data were eliminated. Lastly, relationships among the themes were interpreted and described in connection to participants’ responses.

Several criteria were used to evaluate the trustworthiness of the qualitative data. First, credibility was enhanced, because the first author had spent time in the field and was familiar with the setting. Second, the various perspectives of the research team members, who had different degrees of familiarity with the setting, were included. Third, transferability was assessed, which is a determination of how applicable the findings are to other contexts and is also a way of evaluating trustworthiness of the data [28]. Here, the transcripts were translated to English, and discussion of emerging themes among research team members enriched the interpretation of the data through the balance of perspectives representing different backgrounds and qualitative expertise. These measures enhanced the credibility of the representation of participants’ views presented here. Our study also follows the consolidated criteria for reporting qualitative research (COREQ) recognizing and addressing the importance of the research team, study design, sample size, and analysis and findings [29]. Our detailed description of the study context, selection criteria, data collection, and analytical process is complemented below by quotations from participant interviews, which allow readers to judge the dependability of the analysis and transferability of the findings.

## Results

### Participant demographics

In the sample of 22 adolescents interviewed, ages ranged from 13 to 19, with a mean of 16.6 years. Adolescents were generally evenly represented by gender, with 12 females and ten males. From an educational perspective, three were in primary school, ten were in secondary school, five were vocational education and training authority students, one was in adult education classes, and one was a university student. The remaining two participants had completed primary school, and both were employed. All participants knew their HIV status; 16 were tested at a young age (less than 5 years), and six were tested at age 12 years or older. Age at disclosure of HIV status to adolescents ranged from 10 to 18 years (mean = 11.5).

### Qualitative themes

Several themes emerged from the data: adolescents’ lack of participation in decisions about HIV care and treatment; issues around disclosure of HIV status, such as delays in disclosure to the adolescent and disclosure to other persons and benefits and harms of such disclosures; and factors supporting and interfering with adherence to ART, such as parental support, organizational (clinic) support and problems, and self-stigmatization and shame.

#### Lack of participation in decision making regarding enrollment in care and treatment of HIV

As noted, 16 participants were tested when they were younger than age 5 and did not have the capacity to decide to enroll in care and treatment for their HIV. Decisions about testing and enrollment were made by their parents, mostly mothers. Regardless of who made the decision about enrollment in the CTC, adolescents reported adhering to their medication for their own health benefit, as signified by the quotes below:

I was born with this disease; therefore, I was enrolled when still very young—my mother made the decision. I take medicine by adhering to treatment because I am living with HIV, therefore, I take ART in order to improve my body immunity. (Adolescent # 09).

I was enrolled when very young—my mother decided for me to be tested and enrolled in CTC... I do take medicine in my mother’s room. (Adolescent #01).

Six adolescents were tested and enrolled in the CTC at an older age (12–16 years). This decision was made by their primary care giver. These adolescents reported that they were not involved in the decision-making process and were unhappy about their lack of participation in testing for HIV and then enrollment in the CTC. Even though they were unhappy, they still took their medicine, as noted below.

I was tested at age 12, and I was very sick—hence not involved in the decision to test. After the HIV testing, disclosure was done to my grandfather, who later on informed me. I felt sad in my heart for not being asked. (Adolescent #04).

I was tested when I was 15 years, I was not involved in the decision to be HIV tested despite being in the room. I felt bad because they discussed about my HIV status as if I am not existing in the room….but I did not regard it as a mistake; for me it was a kind of love from my mother because through that I knew my health status that am living with HIV, this helped me to start medication early. I think if I did not start medication early, I would have already passed away. (Adolescent # 03).

This participant also added, “Adolescents have to be given a chance to participate in the decision making for medical care. I do not blame my mother for what she did, but adolescents have to participate in the matters that affect their life.” (Adolescent # 03).

#### Disclosure of HIV status to the adolescent

##### Delays in disclosure leading to poor adherence to ART

Most adolescents reported a delay in disclosure of their HIV status to themselves (disclosure at ages 10–18 years). Disclosure should take place by the time an individual is 7 years old, according to national HIV guidelines [30].

My father gave me information about my health status when I was eleven years old; he told me that by now you are matured person, and you are living with HIV virus from your mother. I was heartbroken because I did not know what was going on, they were hiding this information. (Adolescent # 09).

Some of the adolescents felt that they were HIV infected even before they were informed, as shown by the following quotes:

I was told by my mother when I was twelve years after asking her why am I using medicine for a long time. Through attending clubs, I suspected I may be living with HIV. In clubs we were taught issues that made me know my health status. (Adolescent # 22).

Nondisclosure for some adolescents resulted in poor adherence to medication and deterioration of health status, as signified by the following quote:

I refused to take medicine until I knew why I was taking these medicines; at first I was told to take medicine because they accelerate growth since I was so thin. My health deteriorated after stopping ART. (Adolescent # 11).

#### Disclosure of adolescents’ HIV status to others

##### Benefit of disclosure of HIV status of adolescents to others

Participants revealed that disclosing HIV infection to others, including friends, was helpful, as illustrated by the below quote:

One of my friends at school knows my health status; she helps me when I get sick. I was in a critical health condition when I decided to tell her (Adolescent # 21).

Adolescents living with HIV disclosed their HIV status to teachers and matrons for support to take their medicine appropriately and without missing clinic appointments. Several quotes are provided below.

I told my teachers because I wanted them to know why I was not attending school sometimes; hospital administration wrote a letter and was submitted to school by my brother (Adolescent # 21).

My class teacher was aware about this situation, since he is the one who was responsible to give me permission to attend clinic... My parents informed him about my situation. (Adolescent # 04).

Sometimes adolescents felt that they were compelled to disclose their HIV status due to school regulations and requirements, as shown in the quote below:

Only my form 2 teachers know my health status. It was a day whereby students who had problems, like the deaf, disabled, were explaining their problems, so the teacher said whoever knows that she/he is sick has to report, and being with HIV infection, I have to report. (Adolescent # 01).

##### Harm of disclosure of HIV status of adolescents to others

Some adolescents reported being stigmatized and called names after HIV disclosure to others.

My mother died when I was young, and I am living with my father and stepmother. My stepmother insults me due to HIV infection and the need to go for medicine always. She discloses my HIV status to everyone who comes to our family, without caring for the impact of discrimination to me. I sometimes feel it is better if I was not born. (Adolescent # 09).

One adolescent did not feel comfortable going to university for fear of other university students knowing her HIV status, as shown by the below quote.

… I was selected to join a government University in Dodoma, which is good and cheaper, but I decided to find a private college in Dar es Salaam for fear of colleagues finding out about my HIV status when I have to move to hostel if I have to take medicine in their presence. (Adolescent # 20).

#### Adolescent’s experiences that influenced the decision to adhere to ART

##### Parental support for their adolescents to adhere to ART

Participants raised concerns about their inability to attend the CTC despite understanding the importance of ART due to cost implications, as shown in these quotes:

It is a personal decision to come to clinic to get medicine, counselling on reproductive issues, and other services, but I do not want to come to CTC because of the cost of fare and food. (Adolescent #21).

It costs me because sometimes I wake up without bus fare, sometime when I attend CTC my friends are eating food, but I cannot afford to eat because I do not have money to buy food. (Adolescent # 22).

Adolescents needed their parents’ logistical and financial support, such as bus fare, food, and drinks, to come to the CTC for ART and other services provided, including counselling, as seen in the below quotes:

My parents are supportive of my care, since I was young and suffered HIV. Many parents might have already given up and foregone treatment, but my parents are taking care of me. (Adolescent # 05).

My father is very supportive of my care, my mother died when I was young (Adolescent # 09).

Other relatives also provided support, although sometimes they may fail to provide for needed care, as shown below:

It is the bus fare—if it happens that those whom I am living with have no money, then I have to find other relatives who can help. (Adolescent # 04).

##### Health care organizational factors

Participants reported that health care organizational factors are important to their adherence to ART, including time spent waiting for care, availability of medicine, health education provided at the clinic, adolescents’ HIV clubs (organized groups of HIV-infected adolescents with the aim of encouraging one another), and attitudes of health care workers.

I do not want to come to clinic, it is costly, I need time, there are traffic jams on the way to hospital, and I miss classes at school. (Adolescent # 20).

We delay at the hospital, there is a shortage of medicine; living far away causes me to arrive at the hospital beyond the time for services and means that I do not get medicines. (Adolescent # 11).

Health care workers are coming to work late, hence starting clinic late—therefore, delaying us unnecessarily. (Adolescent # 22).

The challenge that I face is time; sometimes I take medicine late because the person who is distributing cards reports late to clinic. (Adolescent # 15).

Initially clinic was on Saturday, but this year I have to come on Friday; it is difficult to attend clinic on Friday because I have classes at school. (Adolescent # 01).

Sometimes some doctors make annoying language towards patients; we stay for a long time waiting for services while doctors are chatting or eating without bearing in mind that patients have other societal obligations. (Adolescent #07).

##### Individual adolescent factors

Some adolescents reported self-stigmatization and blaming themselves for being HIV infected. This affected their decision to adhere to care and treatment, as shown by the quotes below:

I do hide; I hide myself a lot when taking ART because I do not want anyone to know that I am HIV infected. (Adolescent # 01).

On further inquiry he reported, “I do not want people to stigmatize my mother; she is a petty trader, and if they cannot buy from her, we can suffer.” (Adolescent # 01).

I do not want to attend clinic due to a feeling of shame when going to HIV care and treatment clinic to take medicine. (Adolescent #15).

## Discussion

This is one of the first studies to focus on adolescents’ experience of participating in a care and treatment clinic for HIV in Tanzania. Several ethical concerns were noted. First, adolescents sought participation and some control in decision making about their health and well-being, but many found this difficult to do because such decisions were made before they became aware of their HIV status. Lack of participation made adolescents feel disappointed, but they also understood the importance of being treated. Second, some adolescents felt shame, stigma, and worthlessness associated with their HIV status. Third, delays in disclosure of HIV status to adolescents resulted in some adolescents neglecting ART and a worsening of their health status. Disclosure to others was regarded as both beneficial and harmful.

Individual participation in medical decisions is an important ethical requirement for everyone, including adolescents in low-to middle-income countries [31]. The results of this study, however, show a lack of adolescents’ involvement in decision making about HIV testing as well as enrollment in ART. Guidelines to involve adolescents in their testing decision vary from one practice setting to another [32]. In South Africa, adolescents from age 12 onward can consent to HIV testing [33], while in Tanzania the age of consent for HIV testing is 16 [34]. Most adolescents in our study were tested at a young age and were not able to deliberate about testing for HIV and its implications. Parents make decisions in their children’s best interest, and findings from this study reveal that adolescents appreciated their parents’ love for them. However, our participants also wanted to make decisions for themselves. Despite being HIV tested and enrolled in care in the CTC without their consent, adolescents adhered to ART because they believed it improved their health. Hosek et al. [35] conducted a qualitative inquiry and found similar results with U.S. adolescents, who adhered to their medication because they wanted to stay healthy and alive. Six adolescents in our study were tested between the ages of 12 and 16; however, they were not involved in the decision to test and enroll in care. These adolescents felt that they had been ignored, but they adhered to medication for their own health benefit.

Despite their perception of a lack of involvement in decisions, all adolescents who participated in this study adhered to HIV care and treatment and attended the CTC. Parental support was a key factor in reminding adolescents to attend the clinic; emotional and economic support were also among the issues mentioned by adolescents as a factor in adherence to ART. This finding is similar to findings of a study conducted in Brazil [36]. In that study, adolescents living in a stable family structure had better adherence to medication and were less likely to face challenges, such as stigma and discrimination. This finding is also supported by other studies [37].

Participants also reported feelings of personal shame that they are living with HIV, which resulted in neglecting appointments and in not taking their ART on time. Self-stigmatization and feelings of worthlessness were among the individual factors reported by our study participants as reasons for poor adherence to medication. A similar study conducted with adults in Northern Tanzania reported that stigma and involuntary disclosure (i.e., learning about one’s HIV status accidentally) were associated with poor adherence to ART [38]. Another factor mentioned by adolescents was tight school schedules, which led to forgetting their medicine or even ignoring ART when they were hungry and busy. ART side effects, such as nausea, dizziness, and drowsiness, can affect adolescents in their day-to-day activities; however, adolescents’ life depends on consistent adherence to ART. The adolescents in our study knew that they had no choice to decline medication if they wanted to survive.

Disclosure of HIV status is an important step in adolescents’ adherence to medication and their growth and development [39]. Unfortunately, disclosure of HIV status to adolescents is delayed in many situations [40]. The current study found that some adolescents were left wondering why they were taking medicine daily. For some study participants, nondisclosure resulted in poor adherence to medication and deterioration of their health status. Other studies have had similar findings [19, 41].

Some of our adolescent participants informed their teachers about their HIV status. Such disclosure helped adolescents keep clinic appointments without worries or fears of missing school. Friends also supported sick adolescents when they knew they had HIV, which also improved their adherence to their medication. In some instances, adolescents informed their siblings, who also conferred emotional support whenever they needed it [42].

Disclosure is an ethical dilemma, because despite its benefits it may also lead to stigma and discrimination, which are physically and emotionally harmful for HIV-infected adolescents. Some participants were ostracized by their relatives because of their HIV status. Some participants also reported being bullied and segregated from others. Disclosure to their sexual partners led to broken relationships and fears of not being able to become engaged again. HIV-infected individuals have a duty to warn sexual partners and to give their partner the chance to make an informed choice. But this may result in stigma and discrimination against the HIV-infected individual [43].

The health care facility environment and adolescent friendliness are among the factors that facilitated adolescents’ decision to attend the HIV CTC. This finding is also supported by other studies [44]. Disrespectful providers discouraged CTC attendance. This finding is similar to a finding in a 2014 study in which participants reported clinic friendliness as important to adolescents [45]. This included flexibility in the days and times of the clinic opening that did not collide with school activities. In that study, service providers with good language skills and infrastructure that protected adolescents’ privacy and confidentiality in terms of their HIV status were also important. In the current study, the time that adolescents spent waiting for their service and providers’ attitudes were also among the main motivators for adolescents to come to the hospital clinic.

Adolescents’ HIV clubs have been found to improve adherence to medication, especially for those ages 15–19 [46]. In adolescent clubs, they are taught about secondary HIV infection and prevention of infection by being responsible and not infecting their partner [41]. Some adolescents in our study reported attending such clubs before their HIV status was disclosed to them—that is, some “got the hint” that they were HIV positive before being told. Uninformed adolescents may wonder why no one is telling them. Knowledge of one’s HIV status is challenging to adolescents, and they may become sad and experience self-stigma, as our data show. But after a period of time, they may learn to live with HIV and assume the responsibilities associated with their disease. Adolescents’ HIV clubs are one of the health care organizational factors that helped them adhere to ART. HIV clubs provide services that include reproductive health services, ART adherence counselling, and an entrepreneurship course. This type of support system can help adolescents living with HIV combat their life challenges with hope and enthusiasm. Other studies have shown teen clubs to be helpful in adolescent retention in HIV care and ART [42]. More research on the benefits and challenges of this type of adolescent support is needed and its role in helping adolescents address their concerns.

## Limitations

The study had several limitations. First, the study interviewed only individuals who agreed to participate. This may have created a selection bias, and those who chose not to participate may have had different concerns not readily addressed in this study. These adolescents may have been uncomfortable discussing their views on parental authority versus adolescent autonomy in decision making for HIV services, on HIV disclosure to others and sexual partners, and on health care workers and the hospital environment in general. To address this limitation, however, the adolescents were told that the researcher would keep their information confidential. In addition, the interviews were conducted in a private room where adolescents were comfortable and away from any outside influences. Generalization of findings from this study is limited to the population of interest because it was conducted with adolescents at one hospital clinic in Tanzania. Future research should examine these issues at other types of clinics and with other adolescents, parents, and providers across different regions of the country. Finally, the small sample size also limits generalizability. However, the qualitative nature of the study provides significant insights, and the greatest strength of a qualitative study is the depth and richness of exploration and description it offers [47].

## Conclusion

Disclosure of HIV status to the adolescent is an ethical dilemma in HIV care of adolescents, but it also can have a positive influence on the adolescent’s life in terms of adherence to ART. Knowledge and adherence can allow adolescents to live a life of purpose and to have hope for the future. Further research is needed on how to involve adolescents in their HIV care and to identify what is important to them as they learn to live with a chronic illness. Their voices are critical in developing strategies and guidelines to improve care delivery for this population. More research is also needed on the role of parental support, compared with adolescents’ HIV clubs, in helping adolescents understand their disease, their disease trajectory, and their responsibilities as they age. Parents may perceive that the risk of harm by lack of autonomy may be considered a “smaller harm” when placed in the context of receiving an early diagnosis and treatment; future research should address these ethical issues and how parents weigh the benefits and the risks of their adolescent’s HIV status. Adolescents are the future; we must continue to respect their views and understand their health needs as they live with a chronic condition that can bring many challenges to their life and the relationships that they develop.

## Data Availability

All data generated or analysed during this study are included in this published article.

## Abbreviations

AIDS: Acquired immunodeficiency syndrome
ART: Antiretroviral therapy
CTC: Care and Treatment Centre
NatREC: National Research Ethics Committee
MUHAS: Muhimbili University of Health and Allied Sciences
UNICEF: United Nations Children’s Fund
